# Hepatic Artery Pseudoaneurysm: A Life-Threatening Complication of Liver Transplantation

**DOI:** 10.5334/jbr-btr.970

**Published:** 2015-12-30

**Authors:** Selcuk Parlak, Serap Gulcek, Hatice Kaplanoglu, Levent Altin, Mehmet Deveer, Lale Pasaoglu

**Affiliations:** 1 Ankara Numune Education and Research Hospital, TR; 2 Yildirim Beyazit Education and Research Hospital, TR; 3 Sitki Kocman University Radiology Clinic, TR

**Keywords:** Liver transplantations, Hepatic arteries, Computed Tomography, Multidetector

## Abstract

Hepatic artery pseudoaneurysm is a rare but serious complication following liver transplantation. A 50-year-old male patient with ulcerative colitis, sclerosing cholangitis, and end-stage liver disease underwent right lobe transplantation from a living donor. The patient was hospitalized because of impairment in liver function tests and massive pretibial edema three months after surgery. In color Doppler ultrasound and multidetector computed tomography, a pseudoaneurysm with peripheral large thrombus was detected at the anastomosis site extending anterior to the hepatic artery. The patient died as a result of unstable hemodynamic conditions.

## Introduction

Liver transplantation(LT) is the only effective treatment for end-stage liver disease. Hepatic artery pseudoaneurysm(HAP) is a rare but serious complication following LT [1,2]. It usually presents with nonspecific abdominal pain and/or massive gastrointestinal bleeding, but asymptomatic cases have also been reported [3]. The reported incidence of HAP after LT is 0.27–3%, and the mortality rate is from 69–100%. HAP develops in the arterial anastomosis, and it is usually related to infection or technical failure [4]. Here, we present the multidetector computed tomography (MDCT) and the color Doppler ultrasound (CDUS) findings of a significant HAP case that developed after LT.

## Case

**Figure 1:**
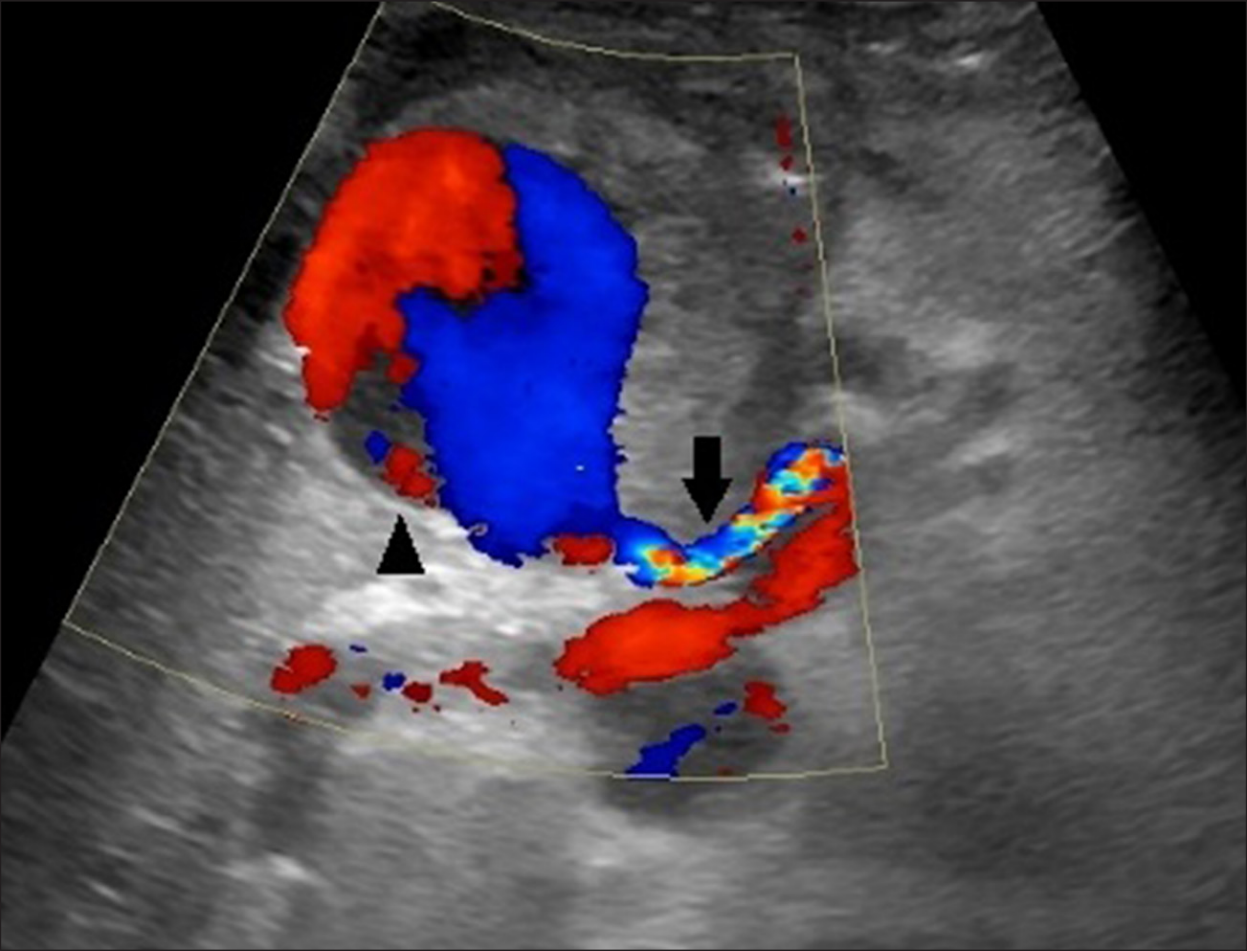
CDUS showing the classic yin-yang flowpattern within the pseudoaneurysm (arrowhead) originating from hepatic artery (arrow). Note that the peripheral partial thrombus in the pseudoaneurysm.

**Figure 2:**
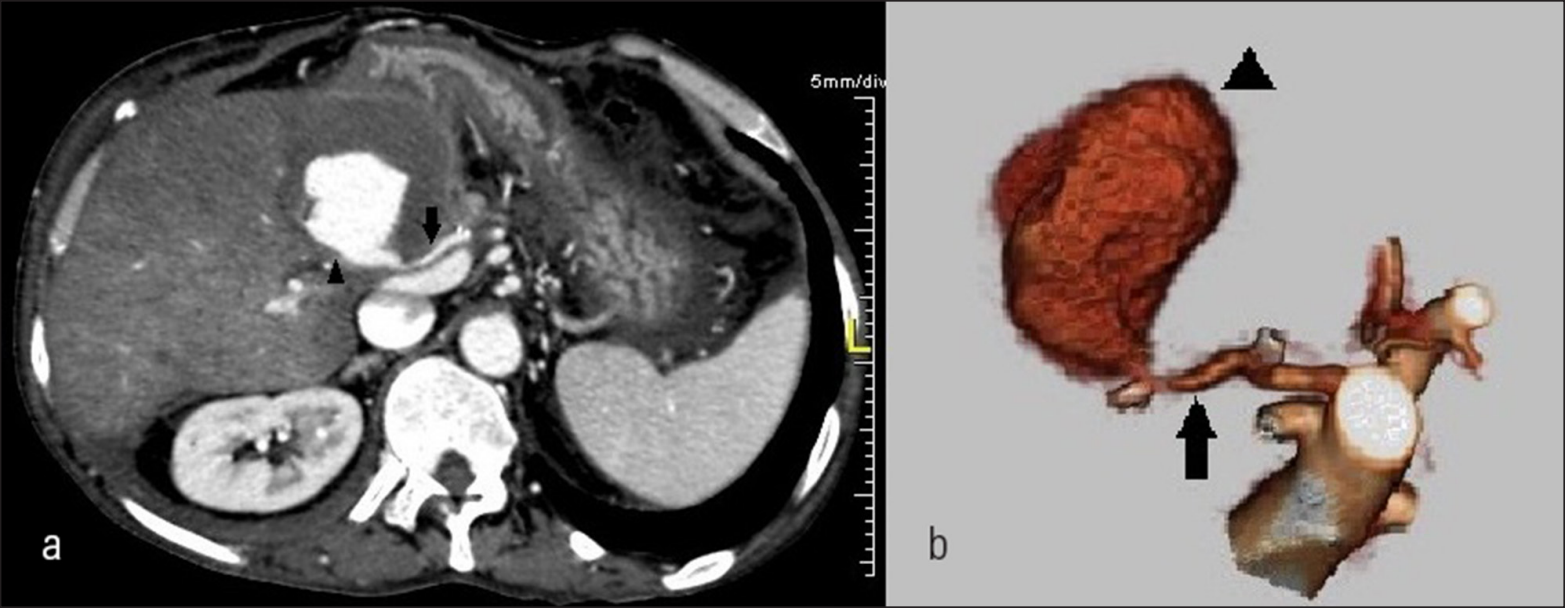
a) MDCT angiogram and b) Volume rendered images show the pseudoaneurysm (arrowhead) originating from hepatic artery (arrow). Hypodense peripheral partial thrombus is also seen around contrastmedia on a).

**Figure 3:**
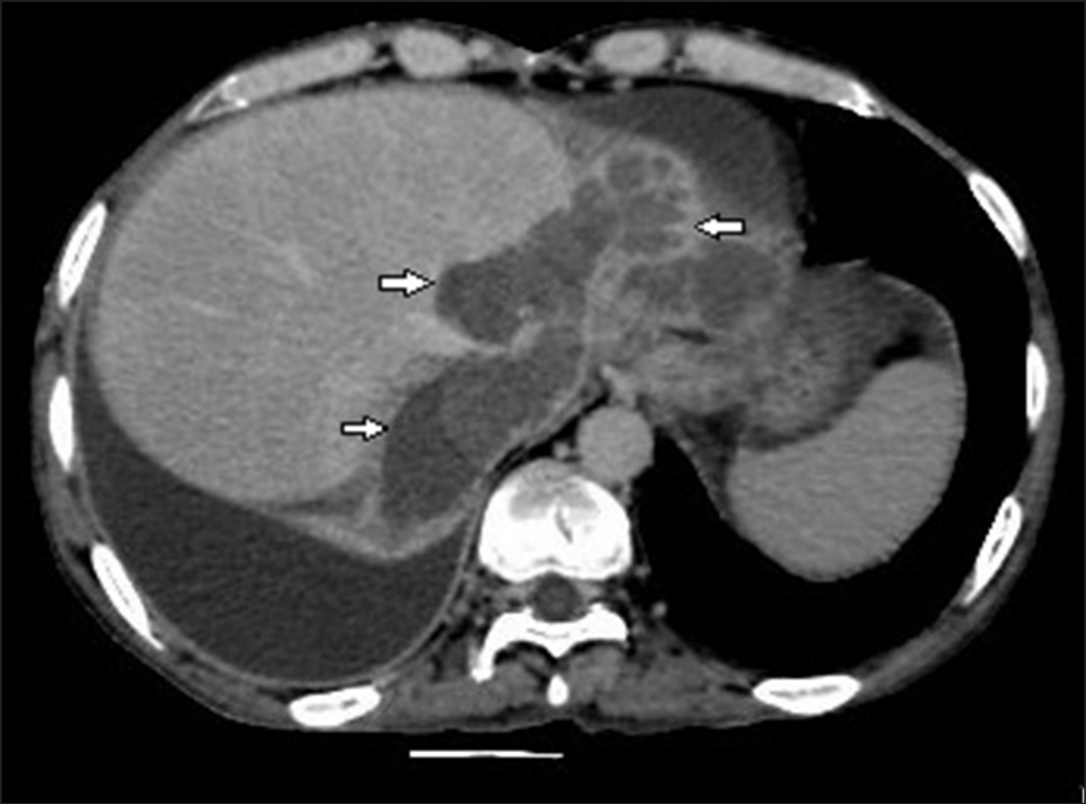
MDCT shows loculated fluid collections thougt as biloma (arrows).

A 50-year-old male patient underwent right lobe liver transplantation from a living donor. The patient was in end-stage liver disease secondary to ulcerative colitis and sclerosing cholangitis. Impairment in liver function tests and massive pretibial edema developed three months after surgery. The patient’s fever was 38,5 °C, and he was hospitalized for treatment. In laboratory analysis, ALT levels were 400 IU/L; AST levels were 300 IU/L; GGT levels were 118 IU/L; direct bilirubin levels were 0,7 mg/dL; and the white blood cell count was 25000/µL (neutrophils 82 %). Meropenem 3 × 1 gram and teicoplanin 1 × 400 mg were started for initial treatment. A pseudoaneurysm measuring 8 × 6.5 cm and having central turbulent flow with peripheral thrombus in CDUS was detected in the portal hilus associated with the hepatic artery (Figure 1). MDCT angiography was applied for a better anatomic orientation; the pseudoaneurysm originated from the hepatic artery anastomosis site and extended anterior to the artery. The central portion of the HAP was filling with contrast media while the peripheral portions were thrombosed (Figure 2). The hepatic artery was 2 mm in diameter, and string-like narrowing was observed distal to the pseudoaneurysm best seen in the arterial phase of the examination. A loculated fluid collection around the pseudoaneurysm extending to the subdiaphragmatic and perihepatic spaces was also observed (Figure 3). In the follow ups, total bilirubin increased to 9,3 mg/dL, direct bilirubin increased to 6,6 mg/dL, and the white blood cell count was 30000/µL. The patient was hemodynamically unstable. Stenting of the hepatic artery was planned, but the patient died before the procedure.

## Discussion

Vascular reconstruction plays a crucial role in the success of orthotopic liver transplantation (OLT). Complications related to reconstruction of the hepatic artery (HA) are the most significant causes of graft loss and mortality [1,3,5,6]. The HA is a small vessel (3–6 mm in adults) with a very fragile intima; therefore, the reconstruction of the artery requires a careful technique. The incidence of arterial complications is between 2 and 25% after OLT [3]. Thrombosis of the HA, stenosis of the HA, HAP, and fistula of the HA are the complications of the HA following OLT [3,6]. The early diagnosis of HAP is of vital importance as it is associated with high mortality due to massive bleeding [1,2,3,7].

According to the site of the aneurysm, HAPs are classified as intrahepatic and extrahepatic [1]. Extrahepatic HAPs are commonly associated with local infection. The agent may be systemic or associated with a subhepatic collection resulting from biliary leak, hepaticojejunostomy, and small bowel perforation [7]. Bilio-enteric anastomosis and post-OLT biliary leaks are thought to be specific risk factors for HAP development [4]. The collection around the HAP in our case was thought to be biliary in origin. Intrahepatic HAPs are related to liver punctures, usually detected incidentally on ultrasound [3,7]. Extrahepatic HAPs originate from the hepatic artery anastomosis site. They can present with bleeding into the peritoneum, retroperitoneum, and gastrointestinal tract [2]. Decreased hemoglobin levels and elevated liver function tests are other presentations [7].

Early diagnosis is important because the detection of a pseudoaneurysm after rupture has high mortality rates [4]. CDUS is usually the first modality in diagnosing vascular complications of OLT. It can be performed bedside with no radiation exposure. But it is sometimes difficult to see the anastomosis site due to a low ultrasonic window. Detection rates of vascular complications are higher on CT. MDCT with CT arteriography is effective in detecting HAP and associated findings [1]. Arteriography is the gold standard technique for the diagnosis of HAP, because pseudoaneurysm and bleeding into the peritoneum and bile ducts can be shown clearly [2].

There are different treatment options for HAP. There are surgical reconstructions (resection, revascularization, ligation), endovascular treatments, such as coil embolization and stent grafting, and retransplantation of the hepatic artery [8]. Ligation of the artery can result in high morbidity and mortality at early stages of OLT. Donor iliac artery and autogenous saphenous vein are used for arterial reconstructions [3]. In recent years, the use of endovascular interventional procedures has increased in the treatment of non-ruptured aneurysms [4].

The diagnosis of HAP has been made easier by the use of color Doppler US and computed tomography. We can achieve high quality images on MDCT angiography. But early diagnosis plays a crucial role in the prognosis of this rare complication, as it may show a fatal course, especially after rupture.

## Competing Interests

The authors declare that they have no competing interests.
